# Benchmarking machine learning and parametric methods for genomic prediction of feed efficiency-related traits in Nellore cattle

**DOI:** 10.1038/s41598-024-57234-4

**Published:** 2024-03-17

**Authors:** Lucio F. M. Mota, Leonardo M. Arikawa, Samuel W. B. Santos, Gerardo A. Fernandes Júnior, Anderson A. C. Alves, Guilherme J. M. Rosa, Maria E. Z. Mercadante, Joslaine N. S. G. Cyrillo, Roberto Carvalheiro, Lucia G. Albuquerque

**Affiliations:** 1 School of Agricultural and Veterinarian Sciences, São Paulo State University (UNESP), Jaboticabal, SP 14884-900 Brazil; 2 Department of Animal and Dairy Sciences, University of Wisconsin, Madison, WI 53706 USA; 3 Institute of Animal Science, Beef Cattle Research Center, Sertãozinho, SP 14174-000 Brazil; 4 National Council for Science and Technological Development, Brasilia, DF 71605-001 Brazil

**Keywords:** Animal breeding, Genomics

## Abstract

Genomic selection (GS) offers a promising opportunity for selecting more efficient animals to use consumed energy for maintenance and growth functions, impacting profitability and environmental sustainability. Here, we compared the prediction accuracy of multi-layer neural network (MLNN) and support vector regression (SVR) against single-trait (STGBLUP), multi-trait genomic best linear unbiased prediction (MTGBLUP), and Bayesian regression (BayesA, BayesB, BayesC, BRR, and BLasso) for feed efficiency (FE) traits. FE-related traits were measured in 1156 Nellore cattle from an experimental breeding program genotyped for ~ 300 K markers after quality control. Prediction accuracy (Acc) was evaluated using a forward validation splitting the dataset based on birth year, considering the phenotypes adjusted for the fixed effects and covariates as pseudo-phenotypes. The MLNN and SVR approaches were trained by randomly splitting the training population into fivefold to select the best hyperparameters. The results show that the machine learning methods (MLNN and SVR) and MTGBLUP outperformed STGBLUP and the Bayesian regression approaches, increasing the Acc by approximately 8.9%, 14.6%, and 13.7% using MLNN, SVR, and MTGBLUP, respectively. Acc for SVR and MTGBLUP were slightly different, ranging from 0.62 to 0.69 and 0.62 to 0.68, respectively, with empirically unbiased for both models (0.97 and 1.09). Our results indicated that SVR and MTGBLUBP approaches were more accurate in predicting FE-related traits than Bayesian regression and STGBLUP and seemed competitive for GS of complex phenotypes with various degrees of inheritance.

## Introduction

Improving feed efficiency (FE) in beef cattle represents a key factor in increasing the production system profitability, as feed accounts for approximately 50–70% of total costs depending on the production system adopted^1^. Using traditional pedigree-based methods, selecting more efficient animals is challenging since accurate selection requires phenotypic data from a large population^2^, and FE-related traits are expensive and difficult to measure. In this situation, genomic selection^3^ represents an important tool to improve the predictive accuracy for these complex traits in beef cattle^4–6^, allowing a reduction in generation interval and, consequently, increasing the genetic gain per year.

Genomic selection (GS) involves a combination of genetic markers and available phenotypic information to accurately predict the genetic merit of young animals^3^, becoming a feasible alternative to the traditional pedigree-based methodology for selecting animals for improved FE in beef cattle populations. The prediction accuracy of GS depends on different factors, including minor allele frequency^7^, marker density^8^, linkage disequilibrium^3^, the relationship between training and validation population^5,9^, heritability of the traits^7,9^, number of genotyped animals^10^, and statistical method^11^. The GS accuracy is particularly sensitive to the genetic background of the target traits since they may present complex genetic architectures. Most GS approaches assume that observed phenotypes are influenced by multiple loci with additive effects distributed throughout the genome. GS uses a large number of markers and methods that perform variable selection or shrinkage are often employed to estimate effect sizes aiming to improve the predictive performance of GS models^12^.

Traditionally, genetic markers (i.e., single nucleotide polymorphisms—SNPs) in GS are exploited by parametric models, such as genomic best linear unbiased prediction (GBLUP). The GBLUP approach assumes that observed phenotypes are influenced by several loci with additive action distributed across the whole genome, which often enhances predictive performance^13^. On the other hand, Bayesian regression methods assign differential weights to markers to better align with the trait's genetic architecture^12,14^. These parametric approaches assume a linear mapping from genotype to phenotypes, ignoring potential nonadditive genetic effects, such as epistasis and dominance^15^. However, it has been suggested that some traits of economic importance may be modulated by more complex genetic architectures^16^, and statistical approaches that accommodate these complex genetic architectures more efficiently could improve the accuracy of GS.

Recently, increased attention has been directed toward statistical methods that offer greater flexibility in modeling complex phenotypes by accommodating nonadditive inheritance more straightforwardly, aiming to improve GS prediction accuracy^17^. Studies have noted that machine learning (ML) techniques provide great flexibility for accommodating complex associations between predictor features and phenotype and can be helpful for GS^4,18–20^. On the other hand, multitrait methods have also been receiving attention in GS due to their ability to appropriately account for the correlation among traits, which helps to increase prediction accuracy and statistical power while simultaneously reducing selection bias^21,22^, especially when the training sample size is small^23^ and for low-heritability traits that are genetically correlated with a high-heritability trait^24^.

To date, there are few comprehensive comparisons of ML algorithms or MTGBLUP against STGBLUP and Bayesian regression methods, especially for FE-related traits in beef cattle. Hence, this study was carried out to compare the predictive ability of the multi-trait GBLUP and ML (multi-layer neural network and support vector machine) against traditional parametric models, namely the standard genomic best linear unbiased prediction (STGBLUP) and Bayesian regression techniques (Bayesian Ridge Regression, Bayesian Lasso, BayesA, BayesB, and BayesC) for FE-related traits in an experimental Nellore cattle population.

## Material and methods

The FE-related traits and genomic information were obtained for 1,156 animals from an experimental breeding program at the Beef Cattle Research Center (Institute of Animal Science – IZ).

### Phenotypic and genotypic information

Animals were from an experimental breeding program at the Beef Cattle Research Center at the Institute of Animal Science (IZ) in Sertãozinho, São Paulo, Brazil. Since the 1980s, the experimental station has maintained three selection herds: Nellore control (NeC) with animals selected for yearling body weight (YBW) with a selection differential close to zero, within birth year and herd, while Nellore Selection (NeS) and Nellore Traditional (NeT) animals are selected for the YBW with a maximum selection differential, also within birth year and herd^25^. In the NeT herd, sires from commercial herds or NeS eventually were used in the breeding season, while the NeC and NeS were closed herds (only sires from the same herd were used in the breeding season), with controlled inbreeding rate by planned matings. In addition, the NeT herd has been selected for lower residual feed intake (RFI) since 2013. In the three herds, the animal selection is based on YBW measured at 378 days of age in young bulls.

The FE-related traits were evaluated on 1156 animals born between 2004 and 2015 in a feeding efficiency trial, in which they were either housed in individual pens (683 animals) or group pens equipped with the GrowSafe feeding system (473 animals), with animals grouped by sex. From those, 146 animals were from the NeC herd (104 young bulls and 42 heifers), 300 from the NeS herd (214 young bulls and 86 heifers), and 710 from the NeT herd (483 young bulls and 227 heifers). Both feeding trials comprised at least 21 days for adaptation to the feedlot diet and management and at least 56 days for the data collection period. The young bull and heifers showed an average age at the end of the feeding trial was 366 ± 27.5 and 384 ± 45.4 days, respectively.

A total of 780 animals were genotyped with the Illumina BovineHD BeadChip assay (770 k, Illumina Inc., San Diego, CA, USA), while 376 animals were genotyped with the GeneSeek Genomic Profiler (GGP Indicus HD, 77 K). The animals genotyped with the GGP chip were imputed to the HD panel using FImpute v.3^26^ with an expected accuracy higher than 0.97. Autosomal SNP markers with a minor allele frequency (MAF) lower than 0.10 and a significant deviation from Hardy–Weinberg equilibrium (*P* ≤ 10^−5^) were removed, and markers and samples with call rate lower than 0.95 were also removed. An MAF lower than 10% was used to remove genetic markers with lower significance and noise information in a stratified population. After this quality control procedure, genotypes from 1,024 animals and 305,128 SNP markers remained for GS analyses. Population substructure was evaluated using a principal component analysis (PCA) based on the genomic relationship matrix using the ade4 R package (Supplementary Figure S1)^27^.

### Feed efficiency-related traits

Animals were weighed without fasting at the beginning and end of the feeding trial, as well as every 14 days during the experimental period. The mixed ration (dry corn grain, corn silage, soybean, urea, and mineral salt) was offered ad libitum and formulated with 67% of total digestible nutrients (TDN) and 13% of crude protein (CP), aiming for an average daily gain (ADG) of 1.1 kg.

The following feed efficiency-related traits were evaluated: ADG, dry matter intake (DMI), feed efficiency (FE), and RFI. In the individual pens, the orts were weighed daily in the morning before the feed delivery to calculate the daily dietary intake. In the group pens, the GrowSafe feeding system automatically recorded the feed intake. Thus, the DMI (expressed as kg/day) was estimated as the feed intake by each animal with subsequent adjustments for dry matter content. ADG was estimated as the slope of the linear regression of body weight (BW) on feeding trial days, and the FE was expressed as the ratio of ADG and DMI. Finally, RFI was calculated within each contemporary group (CG), as the difference between the observed and expected feed intake considering the average metabolic body weight (MBW) and ADG of each animal (Koch et al., 1963) as follows:

$$DMI=CG+ {\beta }_{0}+{\beta }_{1}ADG+{\beta }_{2}MBW+\varepsilon$$

where ${\beta }_{0}$ is the model intercept, ${\beta }_{1}$ and ${\beta }_{2}$ are the linear regression coefficients for $ADG$ and ${MBW=BW}^{0.75}$, respectively, and $\varepsilon$ is the residual of the equation representing the RFI estimate.

The contemporary groups (CG) were defined by sex, year of birth, type of feed trial pen (individual or collective) and selection herd. Phenotypic observations with values outside the interval of ± 3.5 standard deviations below and above the mean of each CG for each trait were excluded, and the number of animals per CG ranged from 10 to 70.

### Estimation of genetic parameters

The (co)variance components and heritability for FE-related traits were estimated considering a multi-trait GBLUP (MTGBLUP) as follows:

$$\mathbf{y}=\mathbf{X}{\varvec{\upbeta}}+\mathbf{Z}\mathbf{a}+\mathbf{e},$$

Where ${\varvec{y}}$ is the matrix of phenotypic FE-related traits (ADG, FE, DMI, and RFI) of dimension Nx4 (⁠N individuals and four traits); ${\varvec{\upbeta}}$ is the vector of fixed effects, linear and quadratic effects of cow age, and linear effect of animal’s age at the beginning of the test; $\mathbf{a}$ is the vector of additive genetic effects (breeding values) of animal, and $\mathbf{e}$ is a vector with the residual terms. The $\mathbf{X}$ and $\mathbf{Z}$ are the incidence matrices related to fixed (**b**) and random effects (**a**), respectively. It was assumed that the random effects of animals and residuals were normally distributed, as $\mathbf{a}\sim {\text{N}}(0,\mathbf{G}\otimes {\mathbf{S}}_{\mathbf{a}}$) and $\mathbf{e}\sim {\text{N}}(0,\mathbf{I}\otimes {\mathbf{S}}_{\mathbf{e}}$), where $\mathbf{G}$ is the additive genomic relationship matrix between genotyped individuals according to VanRaden^28^, $\mathbf{I}$ is an identity matrix, ⊗ is the Kronecker product, and $${\mathbf{S}}_{\mathbf{a}}=\left[\begin{array}{ccc}{\upsigma }_{{\text{a}}1}^{2}& \cdots & {\upsigma }_{\mathrm{a1,4}}\\ \vdots & \ddots & \vdots \\ {\upsigma }_{\mathrm{a1,4}}& \cdots & {\upsigma }_{{\text{a}}4}^{2}\end{array}\right]$$ and $${\mathbf{S}}_{\mathbf{e}}=\left[\begin{array}{ccc}{\upsigma }_{{\text{e}}1}^{2}& \cdots & {\upsigma }_{\mathrm{e1,4}}\\ \vdots & \ddots & \vdots \\ {\upsigma }_{\mathrm{e1,4}}& \cdots & {\upsigma }_{{\text{e}}4}^{2}\end{array}\right]$$ are the additive genetic and residual (co)variance matrices, respectively. The **G** matrix was obtained according to VanRaden^28^: $\mathbf{G}=\frac{\mathbf{M}{\mathbf{M}}^{\mathbf{^{\prime}}}}{2\sum_{{\text{j}}=1}^{{\text{m}}}{{\text{p}}}_{{\text{j}}}\left(1-{{\text{p}}}_{{\text{j}}}\right)}$ where $\mathbf{M}$ is the SNP marker matrix with codes 0, 1, and 2 for genotypes AA, AB, and BB adjusted for allele frequency expressed as $2{{\text{p}}}_{{\text{j}}}$, and ${{\text{p}}}_{{\text{j}}}$ is the frequency of the second allele jth SNP marker.

The analyses were performed using the restricted maximum likelihood (REML) method through airemlf90 software^29^. The predictf90 software^29^ was used to obtain the phenotypes adjusted for the fixed effects and covariates (${{\text{y}}}^{*}={\text{y}}-{\text{X}}\widehat{\upbeta }$). The adjusted phenotypes were used as the response variable in the genomic predictions.

Tthe GEBVs accuracy (${{\text{Acc}}}_{{\text{GEBV}}}$) in the whole population, was calculated based on prediction error variance (PEV) and the genetic variance for each FE-related trait (${\upsigma }_{{\text{a}}}^{2}$) using the following equation^30^: ${\text{Acc}}=1-\sqrt{{\text{PEV}}/{\upsigma }_{{\text{a}}}^{2}}$ .

### Data splitting

A forward validation scheme was applied for computing the prediction accuracies using machine learning and parametric methods, splitting the dataset based on year of birth, with animals born between 2004 and 2013 assigned as the reference population (n = 836) and those born in 2014 and 2015 (n = 188) as the validation set. For ML approaches, we randomly split the training dataset into fivefold to train the models.

### Genomic selection (GS) analyses

#### Parametric methods

##### Single-trait GBLUP (STGBLUP)

Genomic prediction for FE-related traits considering the STGBLUP can be described as follows:

$${\mathbf{y}}^{\mathbf{*}}={\varvec{\upmu}}+\mathbf{Z}\mathbf{a}+\mathbf{e}$$

where ${\mathbf{y}}^{\mathbf{*}}$ is the Nx1 vector of adjusted phenotypic values for FE-related traits, $\upmu$ is the model intercept, $\mathbf{Z}$ is the incidence connecting observations; $\mathbf{a}$ is the vector of predicted values, assumed to follow a normal distribution given by ${\text{N}}(0,{\mathbf{G}}\sigma_{a}^{2}$) and $\mathbf{e}$ is the Nx1 vector of residual values considered normally distributed as ${\text{N}}(0,\mathbf{I}{\upsigma }_{{\text{e}}}^{2}$), in which **I** is an identity matrix, ${\upsigma }_{{\text{e}}}^{2}$ is the residual variance. The STGBLUP model was performed using blupf90 + software^29^.

### Multi-trait GBLUP (MTGBLUP)

Genomic prediction for FE-related traits considering MTGBLUP can be described as follows:

$${\mathbf{y}}^{\mathbf{*}}={\varvec{\upmu}}+\mathbf{Z}\mathbf{a}+\mathbf{e}$$

where ${\mathbf{y}}^{\mathbf{*}}$ is the matrix of adjusted phenotypes of dimension Nx4, $\upmu$ is the trait-specific intercept vector, $\mathbf{Z}$ is the incidence matrix for the random effect; $\mathbf{a}$ is an Nx4 matrix of predicted values, assumed to follow a normal distribution given by ${\text{MVN}}(0,{\mathbf{G}} \otimes {\mathbf{S}}_{{\mathbf{a}}}$) where ${\mathbf{S}}_{\mathbf{a}}$ represents genetic (co)variance matrix for the FE-related traits (4 × 4). The residual effects (**e**) were considered normally distributed as ${\text{MVN}}(0,\mathbf{I}\otimes {\mathbf{S}}_{\mathbf{e}}$) in which **I** is an identity matrix, and ${\mathbf{S}}_{\mathbf{e}}$ is the residual (co)variance matrix for FE-related traits (4 × 4). The MTGBLUP was implemented in the BGLR R package^14^ considering a Bayesian GBLUP with a multivariate Gaussian model with an unstructured (co)variance matrix between traits (${\mathbf{S}}_{\mathbf{a}}$) using Gibbs sampling with 200,000 iterations, including 20,000 samples as burn-in and thinning interval of 5 cycles. Convergence was checked by visual inspection of trace plots and distribution plots of the residual variance.

### Bayesian regression models

Five Bayesian regression models with different priors were used for GS analyses: Bayesian ridge regression (BRR), Bayesian Lasso (BL), BayesA, BayesB, and BayesC. The Bayesian algorithms for GS were implemented using the R package BGLR version 1.09^14^. The BGLR default priors were used for all models, with 5 degrees of freedom (dfu), a scale parameter (S), and π. The Bayesian analyses were performed considering Gibbs sampling chains of 200,000 iterations, with the first 20,000 iterations excluded as burn-in and a sampling interval of 5 cycles. Convergence was checked by visual inspection of trace plots and distribution plots of the residual variance. For Bayesian regression methods, the general model can be described as follows:

$${\mathbf{y}}^{\mathbf{*}}=\upmu +\sum_{{\text{w}}=1}^{{\text{p}}}{{\text{x}}}_{{\text{iw}}}{{\text{u}}}_{{\text{w}}}+{{\text{e}}}_{{\text{i}}}$$

where $\upmu$ is the model intercept; ${{\text{x}}}_{{\text{iw}}}$ is the genotype of the ith animal at locus w (coded as 0, 1, and 2); ${{\text{u}}}_{{\text{w}}}$ is the SNP marker effect (additive) of the w-th SNP (p = 305,128); and ${{\text{e}}}_{{\text{i}}}$ is the residual effect associated with the observation of ith animal, assumed to be normally distributed as $\mathbf{e}\sim {\text{N}}(0,{\mathbf{I}\upsigma }_{{\text{e}}}^{2}$).

The BRR method^14^ assumes a Gaussian prior distribution for the SNP markers (${{\text{u}}}_{{\text{w}}})$, with a common variance ${(\upsigma }_{{\text{u}}}^{2})$ across markers so that ${\text{p}}\left({{\text{u}}}_{1},\dots ,{{\text{u}}}_{{\text{w}}}|{\upsigma }_{{\text{u}}}^{2}\right)=\prod_{{\text{w}}=1}^{{\text{p}}}{\text{N}}({{\text{u}}}_{{\text{w}}}{|0,\upsigma }_{{\text{u}}}^{2})$. The variance of SNP marker effects is assigned a scaled-inverse Chi-squared distribution [${\text{p}}$(${\upsigma }_{{\text{u}}}^{2})={\upchi }^{-2}({\upsigma }_{{\text{u}}}^{2}|{{\text{df}}}_{{\text{u}}},{{\text{S}}}_{{\text{u}}})$], and the residual variance is also assigned a scaled-inverse Chi-squared distribution with degrees of freedom (df_e_) and scale parameters (S_e_).

Bayesian Lasso (BL) regression^31^ used an idea from Tibshirani^32^ to connect the LASSO (least absolute shrinkage and selection operator) method with the Bayesian analysis. In the BL, the source of variation is split into residual term (${\upsigma }_{{\text{e}}}^{2}$) and variation due to SNP markers (${\upsigma }_{{{\text{u}}}_{{\text{w}}}}^{2}$). The prior distribution for the additive effect of the SNP marker $\left[{\text{p}}\left({{\text{u}}}_{{\text{w}}}|{\uptau }_{{\text{j}}}^{2},{\upsigma }_{{\text{e}}}^{2}\right)\right]$ follows a Gaussian distribution with marker-specific prior variance given by ${\text{p}}\left({{\text{u}}}_{{\text{w}}}|{\uptau }_{{\text{j}}}^{2},{\upsigma }_{{\text{e}}}^{2}\right)=\prod_{{\text{w}}=1}^{{\text{p}}}{\text{N}}({{\text{u}}}_{{\text{w}}}\left|0,{\uptau }_{{\text{j}}}^{2}{\upsigma }_{{\text{e}}}^{2}\right)$. This prior distribution leads to marker-specific shrinkage of their effect, whose their extent depends on the variance parameters $\left({\uptau }_{{\text{j}}}^{2}\right)$. The variance parameters $\left({\uptau }_{{\text{j}}}^{2}\right)$ is assigned as exponential independent and identically distributed prior, ${\text{p}}\left( {{\uptau }_{{\text{j}}}^{2} \left| {\uplambda } \right.} \right) = \mathop \prod \limits_{{{\text{j}} = 1}}^{{\text{p}}} {\text{Exp}}\left( {{\uptau }_{{\text{j}}}^{2} \left| {{\uplambda }^{2} } \right.} \right)$ and the square lambda regularization parameter (${\uplambda }^{2})$ follows a Gamma distribution (${\text{p}}\left({\uplambda }^{2}\right)={\text{Gamma}}({\text{r}},\uptheta ))$, where r and $\uptheta$ are the rate and shape parameters, respectively^31^. Thus, the marginal prior for SNP markers is given by a double exponential (DE) distribution as follows: ${\text{p}}\left( {{\text{u}}_{{\text{w}}} \left| {\uplambda } \right.} \right) = \int {{\text{N}}\left( {{\text{u}}_{{\text{w}}} \left| {0,{\uptau }_{{\text{j}}}^{2} ,{\upsigma }_{{\text{e}}}^{2} } \right.} \right){\text{Exp}}\left( {{\uptau }_{{\text{j}}}^{2} \left| {{\uplambda }^{2} } \right.} \right)}$, where the DE distribution places a higher density at zero and thicker tails, inducing stronger shrinkage of estimates for markers with relatively small effect and less shrinkage for markers with substantial effect. The residual variance $({\upsigma }_{{\text{e}}}^{2})$ is specified as a scaled inverse chi-squared prior density, with degrees of freedom df_e_ and scale parameter S_e_.

BayesA method^14,33^ considers Gaussian distribution with null mean as prior for SNP marker effects $({{\text{u}}}_{{\text{w}}})$, and a SNP marker-specific variance (${\upsigma }_{{\text{w}}}^{2}$). The variance associated with each marker effect assumes a scaled inverse chi-square prior distribution, ${\text{p}}\left({\upsigma }_{{\text{w}}}^{2}\right)={\upchi }^{-2}\left({\upsigma }_{{\text{w}}}^{2}|{{\text{df}}}_{{\text{u}}},{{\text{S}}}_{{\text{u}}}^{2}\right)$, with degrees of freedom (${{\text{df}}}_{{\text{u}}}$) and scale parameter (${{\text{S}}}_{{\text{u}}}^{2})$ treated as known^14^. Thus, BayesA places a t-distribution for the marker’s effects, i.e., ${\text{p}}\left({{\text{u}}}_{{\text{w}}}|{{\text{df}}}_{{\text{u}}},{{\text{S}}}^{2}\right)={\text{t}}\left(0,{{\text{df}}}_{{\text{u}}},{{\text{S}}}_{{\text{u}}}^{2}\right)$, providing a thicker-tail distribution compared to the Gaussian, allowing a higher probability of moderate to large SNP effects.

BayesB assumes that a known proportion of SNP markers have a null effect (i.e., a point of mass at zero), and a subset of markers with a non-null effect that follow univariate t-distributions^3,12^, as follows:

$${\text{p}}\left({{\text{u}}}_{{\text{w}}}|{\text{df}},\uppi ,{{\text{df}}}_{{\text{u}}},{S}_{B}^{2}\right)=\left\{\begin{array}{cc}0& \mathrm{with probability \pi }\\ {\text{t}}\left({{\text{u}}}_{{\text{w}}}|{{\text{df}}}_{{\text{u}}},{S}_{B}^{2}\right)& \mathrm{with probability }\left(1-\uppi \right)\end{array}\right.$$

where $\uppi$ is the proportion of SNP markers with null effect, and $1-\uppi$ is the probability of SNP markers with non-null effect contributing to the variability of the FE-related trait^3^. Thus, the prior distribution assigned to SNP with non-null effects is a scaled inverse chi-square distribution.

BayesC method^34^ assumes a spike–slab prior for marker effects, which refers to a mixture distribution comprising a fixed amount with probability $\uppi$ of SNP markers have a null effect, whereas a probability of 1 − π of markers have effects sampled from a normal distribution. The prior distribution is as follows:

$${\text{p}}\left({{\text{u}}}_{{\text{w}}},{\upsigma }_{{\text{w}}}^{2},\uppi \right)=\left\{\prod_{{\text{j}}=1}^{{\text{w}}}\left[\uppi \left({{\text{u}}}_{{\text{w}}}=0\right)+\left(1-\uppi \right){\text{N}}(0,{\upsigma }_{{\text{w}}}^{2})\right]*{\upchi }^{-2}\left({\upsigma }_{{\text{w}}}^{2}|{{{\text{df}}}_{{\text{u}}},\mathrm{ S}}_{{\text{B}}}^{2}\right)*\upbeta (\uppi |{{\text{p}}}_{0},{\uppi }_{0}\right\},$$

Where ${\upsigma }_{{\text{w}}}^{2}$ is the common variance for marker effect, ${{\text{df}}}_{{\text{u}}}$ and ${{\text{S}}}_{{\text{B}}}^{2}$ are the degrees of freedom and scale parameter, respectively, ${{\text{p}}}_{0}$ and ${\uppi }_{0}$[0,1] are the prior shape parameters of the beta distribution.

### Machine learning techniques

Two machine learning (ML) algorithms were applied for genomic prediction: Multi-layer Neural Network (MLNN) and support vector regression (SVR). The ML approaches were used to alleviate the standard assumption adopted in the linear methods, which restrict to additive genetic effects of markers without considering more complex gene action modes. Thus, ML methods are expected to improve predictive accuracy for different target traits. To identify the best combination of hyperparameters (i.e., parameters that must be tuned to control the learning process to obtain a model with optimal performance) in the supervised ML algorithms (MLNN and SVR), we performed a random grid search by splitting the reference population from the forward scheme into five-folds^35^.

### Multi-layer neural network (MLNN)

**Figure 1:**
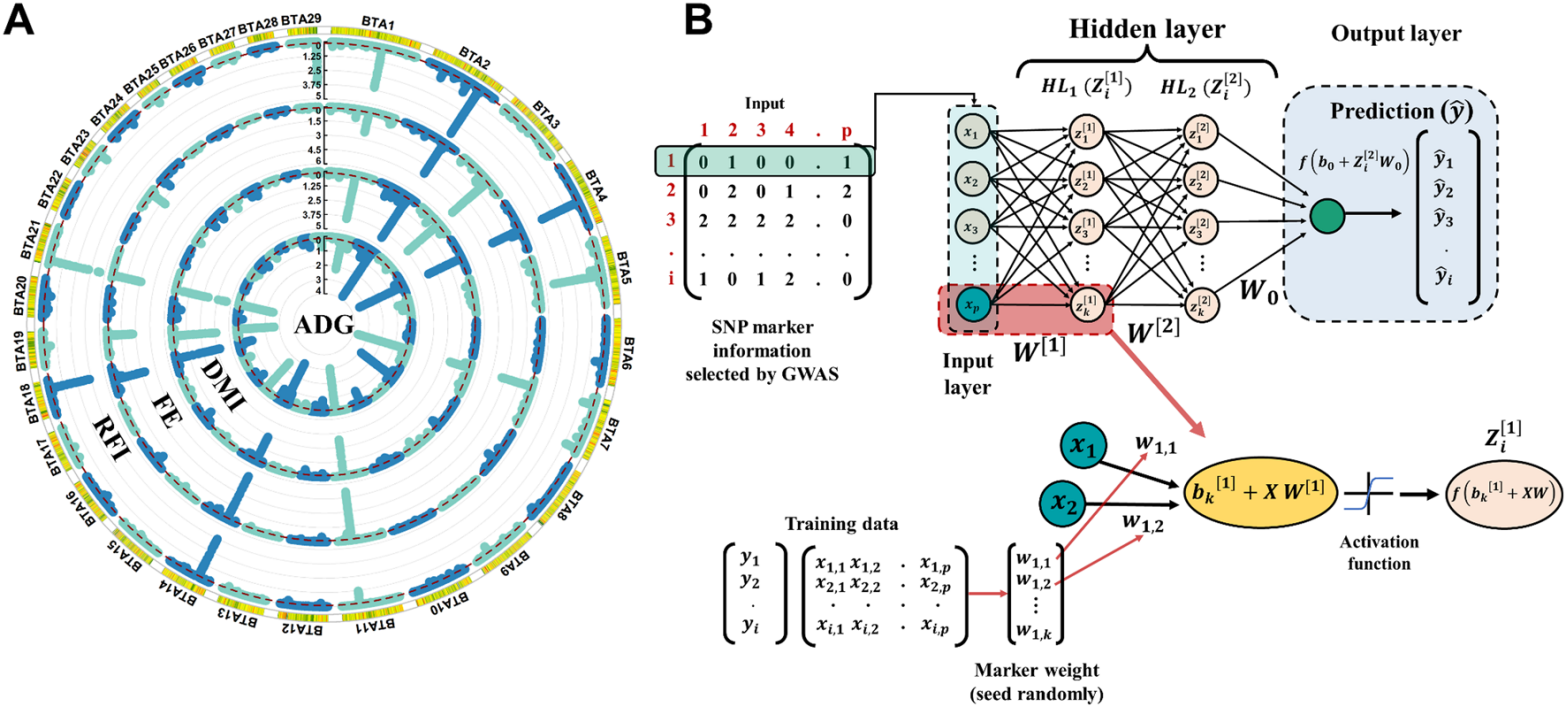
(**A**) Manhattan plot for percentage of genetic variance explained by SNP-marker estimated through multi-trait GWAS in training population to be used as pre-selection strategies for multi-layer neural network. (**B**) General representation of neural networks with two hidden layers used to model nonlinear dependencies between trait and SNP marker information. The input layer ($X={x}_{i,p}$) considered in the neural network refers to the SNP marker information (coded as 0, 1, and 2) of the *ith* animal. The selected node represents the initial weight ($W={w}_{p}$), assigned as random values between -0.5 and 0.5, connecting each input node to the first hidden layer and in the second layer the ${w}_{up}$ refers to the output weight from the first hidden layer, *b* represents the bias which helps to control the values in the activation function. The output ($\widehat{y}$) layer represents a weighted sum of the input features mapped in the second layer.

In MLNN, handling a large genomic dataset, such as 305,128 SNPs, is difficult due to the large number of parameters that need to be estimated, leading to a significant increase in computational demand^36^. Therefore, an SNP pre-selection strategy based on GWAS results in the training population using an MTGBLUP method (Fig. 1A) was used to reduce the number of markers to be considered as input on the MLNN. In addition, this strategy can remove noise information in the genomic data set. In this study, the traits displayed major regions explaining a large percentage of genetic variance, which makes using pre-selected markers useful^37^.

The MLNN model can be described as a two-step regression^38^. The MLNN approach consists of three different layer types: input layer, hidden layer, and output layer. The input layer receives the input data, i.e., SNP markers. The hidden layer contains mapping processing units, commonly called neurons, where each neuron in the hidden layer computes a non-linear function (activation) of the weighted sum of nodes on the previous layer. Finally, the output layer provides the outcomes of the MLNN. Our proposed MLNN architecture comprises two fully connected hidden layers schematically represented in Fig. 1B. The input layer in MLNN considered SNP markers that explained more than 0.125% of the genetic variance for FE-related traits (Fig. 1A; ~ 15 k for ADG and DMI, and ~ 16 k for FE and RFI). The input covariate $X=\{{x}_{p}\}$ contains pre-selected SNP markers (p) with a dimension Nxp (N individuals and p markers). The pre-selected SNP markers are combined with each k neuron (with *k* = 1, …, *Nr*) through the weight vector ($W$) in the hidden layer and then summed with a neuron-specific bias (${b}_{k}$) for computing the linear score for the neuron *k* as:${Z}_{i}^{[1]}=f({{b}_{k}}^{[1]}+X{W}^{[1]})$ (Fig. 1B). Subsequently, this linear score transformed using an activation function $f\left(.\right)$ to map *k* neuron-specific scores and produce the first hidden layer output ($f\left({z}_{1,i}\right)$). In the second-hidden layer, each neuron *k* receives a net input coming from hidden layer 1 as: ${Z}_{i}^{[2]}={{b}_{k}}^{\left[2\right]}+{Z}_{i}^{[1]}{W}^{[2]}$, where ${W}^{[2]}$ represents the weight matrix of dimension k x k (k*—*number of neurons) connecting the ${Z}_{i}^{[1]}$ into the second hidden layer, and ${{b}_{k}}^{\left[2\right]}$ is a bias term in hidden layer 2. Then, the activation function is applied to map the *kth* hidden neuron unit in the second hidden layer and generate the output layer as ${V}_{2,i}=f\left({z}_{2,i}\right)$. In the MLNN, a hyperbolic tangent activation function (${\text{tanh}}\left({\text{x}}\right)={{\text{e}}}^{{\text{x}}}-{{\text{e}}}^{-{\text{x}}}/{{\text{e}}}^{{\text{x}}}+{{\text{e}}}^{-{\text{x}}}$) was adopted in the first and second layers, providing greater flexibility in the MLNN^39^.

The prediction of the adjusted FE-related trait was obtained as follows^38^:

$${\mathbf{y}}^{\mathbf{*}}=\mathbf{f}\left(\mathbf{b}+{\mathbf{V}}_{2,\mathbf{i}}{\mathbf{W}}_{0}\right)+\mathbf{e}$$

where ${\mathbf{y}}^{\mathbf{*}}$ represents the target adjusted feed efficiency-related trait for the *ith* animal; $k$ the number of neurons considered in the model and assumed the same in the first and second layer; ${\mathbf{W}}_{0}$ represents the weight from the *k* neuron in layer 2, $\mathbf{b}$ is related to the bias parameter. The optimal weights used in MLNN were obtained by minimizing the mean square error of prediction in the training subset^40^.

The MLNN model was implemented using the R package *h2o* (https://github.com/h2oai/h2o-3), with the random grid search using the h2o.grid function (https://cran.r-project.org/web/packages/h2o) to determine the number of neurons to maximize the prediction accuracy. We used the training population split into fivefold to assess the best neural network architecture and then apply it in the disjoint validation set^41,42^. We considered a total of 1000 epochs^36^, numbers of neurons ranging from 50 to 2500 with intervals of 100, and applied a dropout ratio of 0.2 and regularization L_1_ and L_2_ parameters as 0.0015 and 0.0005, respectively. In this framework, the MLNN was performed using two hidden layers of neural networks with the number of neurons (*k*) of 750 for ADG, 1035 for DMI, 710 for FE, and 935 for RFI obtained during the training process.

### Support vector regression

Support vector regression (SVR) is a kernel-based supervised learning technique used for regression analysis^43^. In the context of GS, the SVR uses linear models to implement nonlinear regression by mapping the predictor variables (i.e., SNP marker) in the feature space using different kernel functions (linear, polynomial, or radial basis function) to predict the target information, e.g., adjusted phenotype the GS^44^. SVR can map linear or nonlinear relationships between phenotypes and SNP markers depending on the kernel function. The best kernel function mapping genotype to phenotype (linear, polynomial, and radial basis) was determined using the training subset split into fivefold. The radial basis function (RBF) was chosen as it outperformed the linear and polynomial (degree equal 2) kernels in the training process, increasing 8.25% in predictive ability and showing the lowest MSE.

The general model for SVR using a RBF function can be described as^38,45^: ${\mathbf{y}}_{\mathbf{i}}^{\mathbf{*}}=\mathbf{b}+\mathbf{h}{\left(\mathbf{m}\right)}^{\mathbf{T}}\mathbf{w}+\mathbf{e}$**,** where $\mathbf{h}{\left(\mathbf{m}\right)}^{\mathbf{T}}$ represents the kernel radial basis function used to transform the original predictor variables, i.e. SNP marker information (${\text{m}}$), $b$ denotes the model bias, and $w$ represents the unknown regression weight vector. In the SVR, the learn function $\mathbf{h}{\left(\mathbf{m}\right)}^{\mathbf{T}}$ was given by minimizing the loss function. The SVR was fitted using an epsilon-support vector regression that ignores residual absolute value ($\left|{y}_{i}^{*}-{\widehat{y}}_{i}^{*}\right|$) smaller than some constant (ε) and penalize larger residuals^46^.

The kernel RBF function considered in the SVR follows the form: $\mathbf{h}{\left(\mathbf{m}\right)}^{\mathbf{T}}=\mathbf{exp}\left(-{\varvec{\upgamma}}{\Vert {\mathbf{m}}_{\mathbf{i}}-{\mathbf{m}}_{\mathbf{j}}\Vert }^{2}\right)$, where the ${\varvec{\upgamma}}$ is a gamma parameter to quantity the shapes of the kernel functions, $m$ and ${m}_{i}$ are the vectors of predictor variables for labels *i* and *j*. The main parameters in SVR are the cost parameter (${\text{C}}$), gamma parameter (${\varvec{\upgamma}}$), and epsilon ($\upepsilon$). The parameters ${\text{C}}$ and $\upepsilon$ were defined using the training data set information as proposed by Cherkasky and Ma^47^: ${\text{C}}={\text{max}}\left(\left|\overline{{{\text{y}} }^{*}}+3{\upsigma }_{{{\text{y}}}^{*}}\right|,\left|\overline{{{\text{y}} }^{*}}-3{\upsigma }_{{{\text{y}}}^{*}}\right|\right)$ and $\upepsilon =3{\upsigma }_{{{\text{y}}}^{*}}\left(\sqrt{{\text{ln}}\left({\text{n}}\right)/{\text{n}}}\right)$, in which the $\overline{{{\text{y}} }^{*}}$ and ${\upsigma }_{{{\text{y}}}^{*}}$ are the mean and the standard deviation of the adjusted FE-related traits on the training population, and n represents the number of animals in the training set. The gamma (γ) was determined through a random search of values varying from 0 to 5, using the training folder split into fivefold. The better-trained SVR model considered the γ parameter of 2.097 for ADG, 0.3847 for DMI, 0.225 for FE, and 1.075 for RFI. The SVR was implemented using the e1071 R package^48^.

### Predictive ability

Prediction accuracy (acc) of the different statistical approaches was assessed by Pearson’s correlation between adjusted phenotypes (${{\text{y}}}^{*}$) and their predicted values (${\widehat{{\text{y}}}}_{{\text{i}}}^{*}$) on the validation set, and root mean squared error (RMSE). The prediction bias was assessed using the slope of the linear regression of ${\widehat{y}}_{i}^{*}$ on ${{\text{y}}}^{*}$, for each model. The Hotelling-Williams test^49^ was used to assess the significance level of the difference in the predictive ability of Bayesian methods (BayesA, BayesB, BayesC, BL, and BRR), MTGBLUP, and machine learning (MLNN and SVR) against STGBLUP. The similarity between the predictive performance of the different models was assessed using Ward’s hierarchical clustering method with an Euclidian distance analysis. The relative difference (RD) in the predictive ability was measured as ${\text{RD}}=\frac{({{\text{r}}}_{{\text{m}}}-{{\text{r}}}_{{\text{STGBLUP}}})}{{{\text{r}}}_{{\text{STGBLUP}}}}\times 100$, where ${{\text{r}}}_{{\text{m}}}$ represents the acc of each alternative approach (SVR, MLNN, and MTGBLUP, or Bayesian regression models—BayesA, BayesB, BayesC, BL, and BRR), and ${{\text{r}}}_{{\text{STGBLUP}}}$ is the predictive ability obtained using the STGBLUP method.

### Ethical approval

The animal procedures and data sampling presented in this study were approved and performed following the Animal Care and Ethical Committee recommendations of the São Paulo State University (UNESP), School of Agricultural and Veterinary Science (protocol number 18.340/16).

## Results

### Description of phenotypic and genotypic information

Principal component (PC) analysis identified some genetic stratification among the selection herds in the population, with the first two PCs explaining 3.27% of genetic variation (Additional File1: Figure S1). As expected, two selection herds in the IZ population (NeS and NeT) exhibited more genetic similarity, whereas the NeC herd was a completely separated cluster. The heritability estimates for FE-related traits were moderate, ranging from 0.21 ± 0.029 for FE to 0.40 ± 0.039 for DMI (Table 1). These heritability estimates indicated that a substantial component of the phenotypic variation in FE-related traits is due to additive genetic effects. The genetic correlations between FE-related traits were from low (*r* = 0.22 for RFI x ADG) to high moderate (*r* = 0.66 for RFI x DMI), and the FE showed a negative correlation with DMI and RFI (Supplementary Figure S2).

**Table 1:** Descriptive statistics for phenotype and adjust phenotype, estimates of additive genetic variances (${\sigma }_{a}^{2}$), heritabilities (${h}^{2}$) with its respective standard error (SE) and the avarege accuracy of genomic breeding value (${Acc}_{GEBV}$) of all animals with standard deviation (SD) for feed efficiency-related traits, using the multi-trait GBLUP.

Trait	Phenotype				Adjusted phenotype ($\widehat{y}$)				${\sigma }_{a}^{2}$± SE	${\sigma }_{p}^{2}$± SE	${h}^{2}$± SE	${Acc}_{GEBV}$± SD
	Mean	SD	Min	Max	Mean	SD	Min	Max				
ADG (kg/day)	1.04	0.25	0.39	1.72	0.43	0.14	− 0.09	0.87	0.010 ± 0.002	0.30 ± 0.056	0.33 ± 0.042	0.86 ± 0.05
DMI (kg/day)	7.29	1.52	3.00	12.01	3.13	1.15	− 0.77	6.65	0.32 ± 0.033	0.79 ± 0.059	0.40 ± 0.039	0.85 ± 0.05
FE	0.15	0.03	0.06	0.27	− 0.04	0.02	− 0.12	0.05	0.001 ± 0.0004	0.005 ± 0.0006	0.21 ± 0.029	0.82 ± 0.04
RFI (kg/day)	− 0.02	0.59	− 2.37	3.74	− 1.77	0.68	− 3.85	1.88	0.27 ± 0.05	0.96 ± 0.075	0.28 ± 0.022	0.81 ± 0.06

ADG: average daily gain, FE: feed efficiency, RFI: residual feed intake, DMI: dry matter intake and Adjusted phenotype: phenotype information corrected for fixed effects considered in the model.

### Predictive ability of GS approaches

Prediction accuracy comparison used GBLUP as the benchmark against ML methods (SVR and MLNN), MTGBLUP, and Bayesian regression techniques (BayesA, BayesB, BayesC, BRR, and BL) (Table 2). The prediction accuracy for forward validation is shown in Table 2. The predictive correlation obtained for the various models varied from moderate to high, ranging from 0.53 (BayesA) to 0.67 (SVR) for ADG, 0.54 (BayesA) to 0.62 (MTGBLUP and SVR) for DMI, 0.53 (STGBLUP) to 0.64 (MTGBLUP and SVR) for FE, and 0.62 (STGBLUP) to 0.69 (SVR) for RFI (Table 2). The accuracy obtained for the various models was directly proportional to the trait heritability (Supplementary Figure S3). Overall, the ML techniques (MLNN and SVR) and multi-trait GBLUP resulted in greater prediction accuracies against the standard approaches for GS, i.e., STGBLUP and Bayesian approaches (Table 2).

**Table 2:** Accuracies of genomic prediction and root mean square error (RMSE) assessed for feed efficiency-related traits in testing animals using a forward validation scheme obtained with different parametric and machine learning methods.

Trait^1^	Model fit	Genomic prediction approaches^2^								
		STGBLUP	BayesA	BayesB	BayesC	BL	BRR	MTGBLUP	MLNN	SVR
ADG (kg/day)	*r—training*	0.88	0.96	0.89	0.93	0.95	0.91	0.87	0.88	0.86
	Accuracy	0.58	0.53*	0.59	0.53*	0.54*	0.59	0.66***	0.62**	0.67***
	RMSE	0.061	0.063	0.061	0.062	0.071	0.061	0.048	0.054	0.047
DMI (kg/day)	*r—training*	0.86	0.94	0.92	0.92	0.9	0.87	0.83	0.85	0.83
	Accuracy	0.56	0.54*	0.57	0.55	0.56	0.57	0.62***	0.60***	0.62***
	RMSE	0.365	0.371	0.366	0.373	0.444	0.365	0.276	0.327	0.271
FE	*r—training*	0.95	0.97	0.94	0.9	0.89	0.88	0.84	0.86	0.83
	Accuracy	0.53	0.54	0.56	0.54	0.57*	0.56*	0.64***	0.61***	0.64***
	RMSE	0.007	0.007	0.007	0.006	0.008	0.006	0.0051	0.006	0.0049
RFI (kg/day)	*r—training*	0.89	0.91	0.93	0.9	0.92	0.89	0.86	0.89	0.86
	Accuracy	0.62	0.63	0.64	0.62	0.63	0.63	0.68***	0.66*	0.69***
	RMSE	0.231	0.235	0.23	0.235	0.284	0.233	0.174	0.209	0.167

^1^ADG: average daily gain, DMI: dry matter intake, FE: feed efficiency, and RFI: residual feed intake, *r*: *training*—$cor({y}^{*},{\widehat{{\text{y}}}}_{{\text{i}}}^{*})$ in training population, Accuracy—*c* $or({y}^{*},{\widehat{y}}_{i}^{*})$.

^2^STGBLUP: single trait GBLUP, BayesA: Bayesian A, BayesB: Bayesian B, BayesC: Bayesian C, BL: Bayesian Lasso, BRR: Bayesian ridge regression, MTGBLUP: multi-trait GBLUP, MLNN: Multi-layer neural networks, and SVR: support vector machine regression using a radial basis kernel. Statistically significant differences between the predictive ability of each method across the herds compared to the standard model (STGBLUP) were *p-value < 0.05, ** p-value < 0.01 and ***p-value < 0.005.

Comparing statistical approaches, the SVR and MTGBLUP had a significantly higher prediction accuracy (*p-value* < 0.05), followed by MLNN, than those obtained by STGBLUP (Table 2). However, the accuracy obtained with BayesA and BayesC was lower, with values ranging from 0.53 for ADG to 0.63 for RFI (Table 2). In contrast, the Bayesian methods statistically underperformed the STGBLUP for ADG (BayesA, BayesC, and BL) and DMI (BayesA and BayesC), while no significant differences in accuracy were observed between Bayesian methods and STGBLUP for FE and RFI (Table 2).

**Figure 2:**
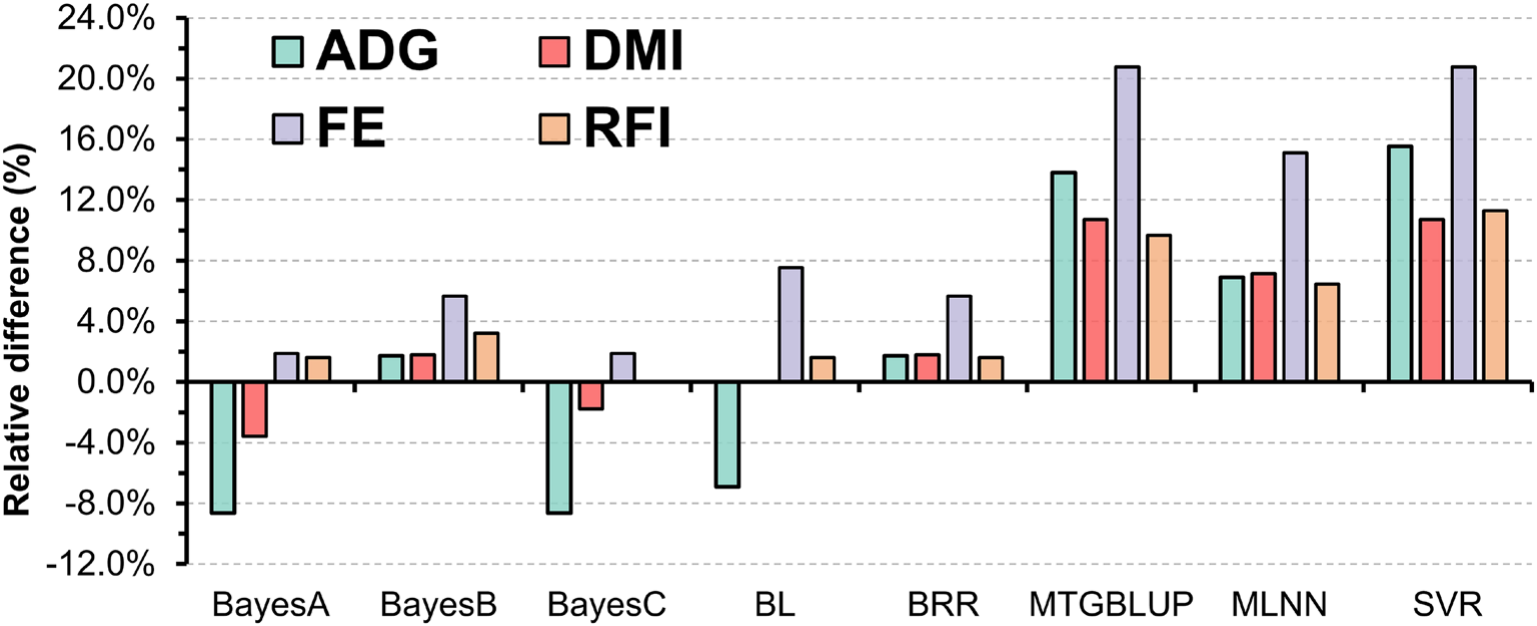
Relative difference in predictive ability using Bayesian methods (BayesA—Bayesian A, BayesB—Bayesian B, BayesC—Bayesian C, BL—Bayesian Lasso and BRR—Bayesian ridge regression), multi-trait GBLUP (MTGBLUP), and machine learning (MLNN—Multi-layer neural networks, and SVR—support vector machine regression) against to single-trait GBLUP (STGBLUP).

Relative difference (RD) between machine learning, MTGBLUP, and Bayesian regression compared with STGBLUP (Fig. 2) indicated an increase in accuracy of about 14.6% for SVR, 13.7% for MTGBLUP and 8.9% for MLNN, compared with the STGBLUP approach. On the other hand, BayesB and BRR models achieved only a slight increment in accuracy, 3.1% and 2.7%, respectively, while predictive accuracies for the FE-related traits were reduced on average by 2.2% with BayesA and 2.1% with BayesC (Fig. 2). In general, the alternative approaches (MLNN, SVR, and MTGBLUP) increased predictive accuracies for all traits, with the highest RD for FE (20.8% using MTGBLUP and SVR, and 15.1% with MLNN) and smallest RD for RFI (9.7% for MTGBLUP, 6.5% for MLNN and 11.3% for SVR; Fig. 2).

### Bias and residual parameters of the GS approach

**Figure 3:**
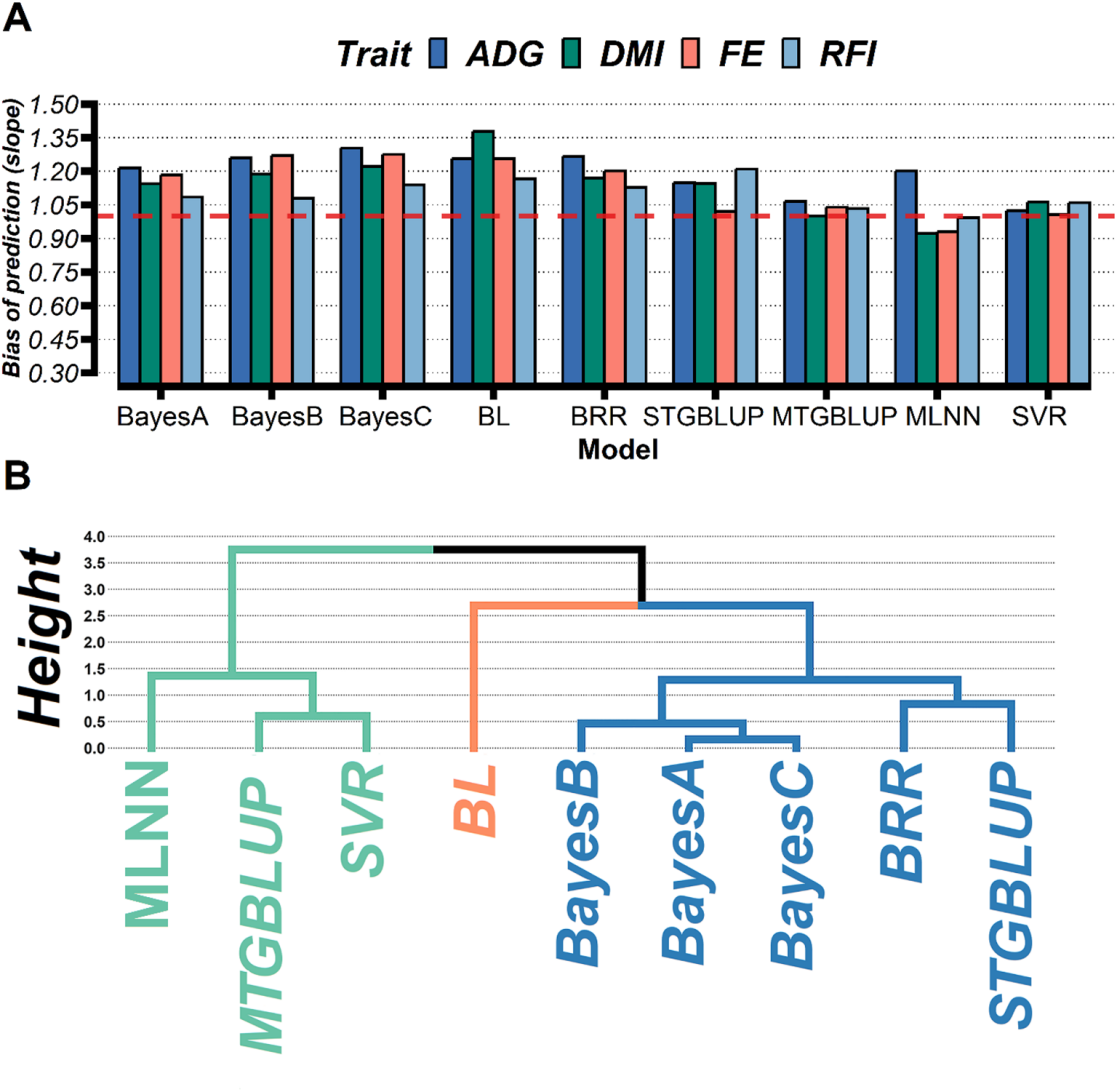
Barplot of the bias of prediction determined as the slope of the linear regression of predicted adjusted phenotype on adjusted phenotype for feed efficiency-related trait using different genomic prediction approaches (**A**) and Ward’s hierarchical clustering of models based on predictive ability and bias for each model (**B**).

The slope coefficient of regression of the predicted and observed (adjusted) phenotype was used to assess the extent of prediction bias (Fig. 3A). Unbiased GS approaches are expected to show a regression slope near 1. The results show that the slope coefficients differed significantly from 1 for the Bayesian methods (BayesA, BayesB, BayesC, BRR, and BL) and STGBLUP (Fig. 3A), so the predictions were empirically biased for these models. On the other hand, slope coefficients for SVR and MTGBLUP differed only slightly from 1, so the predictions were empirically unbiased for these approaches, while MLNN showed biased predictions only for ADG (Fig. 3A).

Using a hierarchical cluster based on accuracy and slope regressions across traits, we observed that alternative approaches (MTGBLUP, MLNN, and SVR) were similar to each other but different from Bayesian regression methods and STGBLUP (Fig. 3B). However, while Bayesian models and STGBLUP tended to be similar, while Bayesian Lasso (BL) showed a greater distance from the others due to its lower predictive ability and highest slope regressions (Fig. 3B).

**Figure 4:**
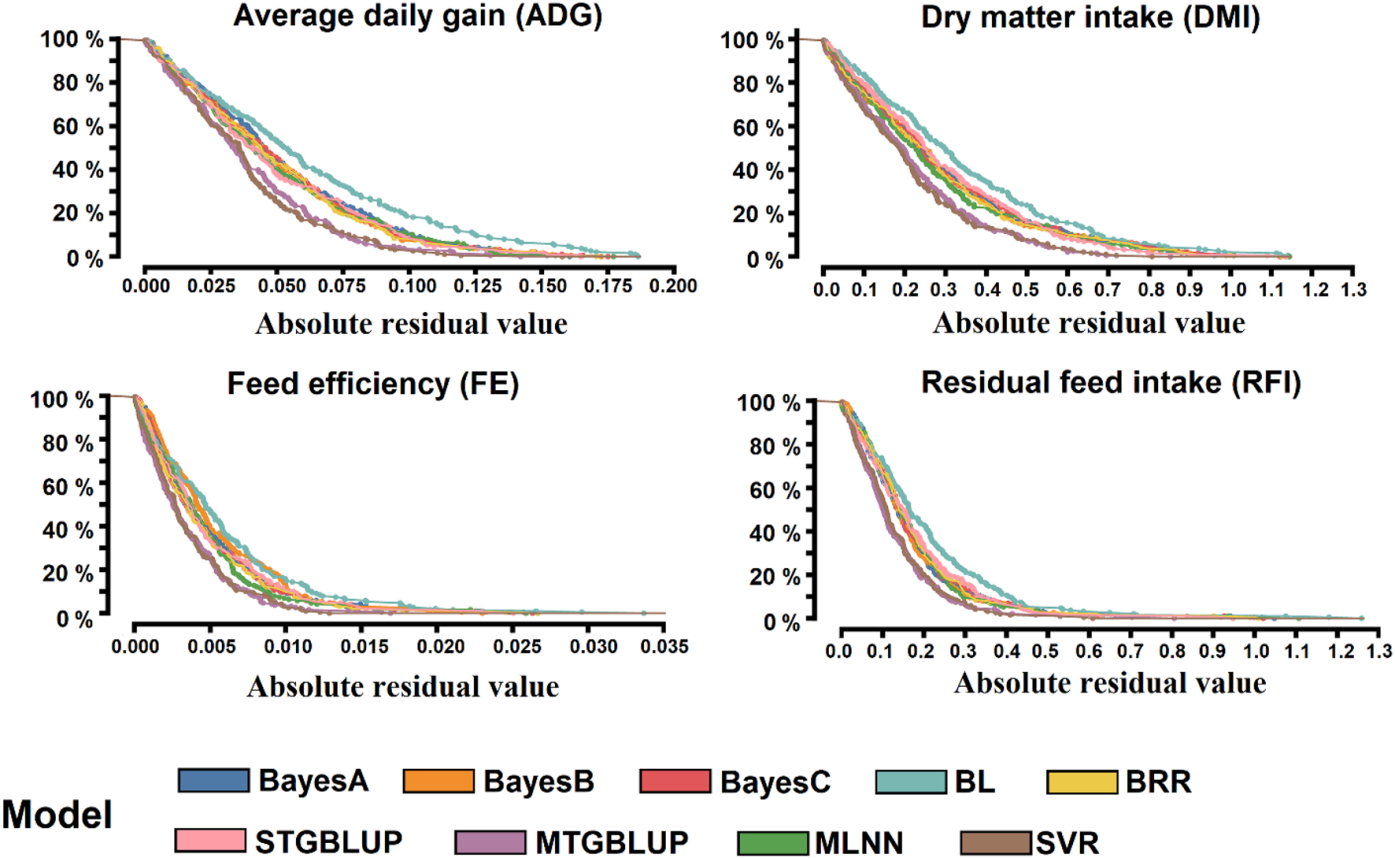
Residual diagnostics comparison for forward validation assessed by the absolute residual value for machine learning (SVR and MLNN) and parametric approaches (BayesA, BayesB, BayesC, BL, BRR, STGBLUP, and MTGBLUP) the genomic prediction approach for the feed efficiency-related traits (ADG, DMI, FE, and RFI) in Nellore cattle.

Assessment of prediction quality through RMSE indicated that ML techniques and MTGBLUP led to considerable reductions in the predictive error compared to STGBLUP (Table 2). Concerning RMSE, the alternative approaches (MTGBLUP, MLNN, and SVR) had the best performance for all traits, with a higher reduction in RMSE compared to STGBLUP and Bayesian regression, varying from 21 to 29% with MTGBLUP, 10% to 14% with MLNN, and 21% to 30% with SVR (Table 2). On the other hand, BL had the highest RMSE value compared with the other approaches, which might be caused by a more biased prediction (Table 2). Comparing the residual values by the probability distribution indicated that SVR and MTGBLUP had a higher probability of lower error than any other approach (Fig. 4). The BL approach showed the highest probability of large residual values, suggesting more biased predictions (Fig. 4).

## Discussion

Genomic prediction of complex traits has been based mainly on parametric methods, which generally consider only additive genetic effects. However, recently, alternative models such as ML or MTGBLUP have been considered as promising approaches for GS due to the potential to capture nonadditive SNP effects for ML or to leverage the genetic correlation between traits for MTGBLUP, and hence improving prediction accuracy^4,18,50,51^. This study compared the prediction accuracy of commonly applied parametric methods (STGBLUP and Bayes regression) with MTGBLUP and nonparametric ML methods (MLNN and SVR) for FE-related traits. We used adjusted phenotypes as the target variables to reduce any noise from contemporary group effects in the phenotype while considering all gene actions. Our results show that the nonlinear prediction methods (MLNN and SVR) and MTGBLUP outperform the other methods for FE-related traits in Nellore cattle (Table 2). However, when the GEBV is used as the target variable in a breeding context, models accounting for exclusively additive effects attributed to markers generally perform better in traits with purely additive inheritance^15^. Nevertheless, Liang et al.^52^ found that combining three machine learning approaches using a stacking ensemble learning framework can accurately predict GEBV for different traits with varying genetic architecture in beef and dairy cattle. Huang and Mackay^53^ have suggested that the estimated additive variance resulting from the similarity between relatives may include a portion of the nonadditive variance. This could explain why nonlinear methods perform better, even if the target variable is expected to depend only on additive effects (GEBV). Depending on the complexity of the trait and the prediction scenario, more sophisticated models help to increase the prediction accuracy. In this situation, using simple models may miss essential data features and cause losses of prediction accuracy for complex traits, whereas nonlinear models usually give better prediction accuracy^19^.

Previous studies reported prediction accuracies of GS for FE-related traits in Nellore cattle ranging from 0.06 to 0.54, obtained with single-step GBLUP and BayesCπ^6^. Our results indicated moderate prediction accuracy and higher than those obtained in other studies for FE-related traits^6,54,55^. In this study, an important factor leading to increased prediction accuracy is the relationship between the reference and testing population (Supplementary Figure S4), which has been pointed out as an important factor to enhance prediction accuracy^5,6,55–57^.

The usefulness of the statistical methods for GS depends on the extent to which these approaches are able to cope with the genetic architecture underlying the target trait and handle the problems associated with SNPs in high linkage disequilibrium^38^. Performing GS using models with less strong assumptions regarding the SNP effects (MLNN and SVR) or considering the correlation structures between the traits (MTGBLUP) outperformed the other models (STGBLUP and Bayesian regression). It has been reported that ML approaches lead to more accurate predictions when the target traits are affected by nonadditive effects due to their ability to account for these complex effects without explicit parametric representation in the models^58^.

The MLNN captures interactions between SNP markers and allows weight differentially in each neuron, reflecting its importance for output prediction^17,59^. On the other hand, the SVR approach uses a RBF to assign flexibility to markers, attempting to match correctly their contributions to the variation of the target trait^60,61^. Although both the SVR and MLNN methods potentially capture nonadditive relationships between phenotypes and SNP markers, SVR using RBF shows advantages over MLNN in terms of more flexibility to handle a smaller number of animals and a larger number of genetic markers; MLNN requires a larger training population to learn from the input data satisfactorily provide accurate predictions with complex genetic architecture of traits^17,62^.

Another factor that could explain the slight differences in results among the approaches is that FE-related traits may be indeed affected by several QTLs with minor effects distributed across the genome^63,64^. This may have favored the models based on the infinitesimal assumption (BRR and STGBLUP) and limited the performance of the Bayesian models, leading to roughly equivalent prediction accuracies. The usefulness of the Bayesian regression approaches, which perform variable selection and/or shrinkage of markers, occurs when few QTLs with major effects explain a large proportion of the trait genetic variance^10,65,66^. Several studies have also reported that these traditional parametric models, with conceptual differences in prior assumptions on marker effects, showed similar or slight differences in predictive ability compared to STGBLUP^4,67–69^.

Statistical methods capturing additive and epistatic effects have recently been reported to perform better than a simple additive model for the genomic prediction of traits with complex inheritance^70,71^. In order to take advantage of nonlinear relations between genomic information and phenotypes, nonparametric methods as machine learning techniques without prior knowledge about the distribution of the SNP marker effects have been exploited^18,20,51,72^. These methods can potentially capture nonadditive marker effects, improving the prediction quality compared to parametric methods^73^. Our results showed that machine learning techniques (MLNN and SVR) and MTGBLUP outperformed conventional methods (STGBLUP and Bayesian models) in predictive ability with unbiased predictions for the FE-related traits (Table 2 and Fig. 3), potentially capturing nonadditive genetic effects. Other studies have also shown that SVR, MLNN, and multi-trait GBLUP are promising approaches capable of outperforming traditional parametric methods commonly used for complex traits controlled by many genes with possible epistatic and pleiotropic effects^4,20,50,74–76^.

The performance of the MLNN approach significantly improved the prediction accuracy by 9.8% and reduced the RMSE by 11% on average, compared to STGBLUP (Table 2 and Fig. 2), indicating its potential usefulness for genome-enabled prediction of FE-related traits in Nellore cattle. These results agree with other studies that observed a reasonable increment in prediction accuracy, although there was no consistent evidence indicating that MLNN can outperform linear models^71,77–79^. The relative performance of MLNN vs. traditional methods depends on the association between the complexity of the genetic architecture of the target trait and the MLNN architecture. Bellot et al.^80^ observed that deep learning techniques such as Multi-layer Perceptrons and Convolutional Neural Networks exhibited a slight reduction in predictive ability compared to Bayesian regression methods (BayesB and BRR) for traits with an additive and polygenic genetic background in humans.

Based on the overall predictive ability, the MTGBLUP and SVR methods outperformed all the other methods (MLNN, S-TGBLUP, and Bayesian regressions). A favorable attribute of SVR algorithms for GS is related to their ability to detect the most informative SNP markers associated with the target phenotypes and use these SNPs to make predictions^46^. According to Yao et al.^74^, machine learning techniques are useful for increasing the accuracy of genomic prediction for RFI evaluated in a small reference population size. In this sense, SVR offers advantages as a robust algorithm in dealing with a large number of predictors, i.e., SNP markers. Overall, SVR increased predictive ability, on average, by 16% (ranging from 11 to 23%; Fig. 2) and led to reductions in RMSE of about 28% for FE-related traits compared to STGBLUP (Table 2). This improvement in predictive ability can be explained by the fact that this method captures non-linear effects through RBF, and has flexibility to deal with noisy variables using appropriate cost functions. Besides, SVR can account for complex epistatic genetic effects without explicitly modeling them, improving the prediction accuracy relative to traditional parametric methods^47^.

Predictions from MTGBLUP were more accurate than those from STGBLUP and Bayesian regression models, highlighting the potential of considering correlated traits in genomic selection (Supplementary Figure S2). From a theoretical point of view, the genetic correlation across traits can arise mainly from the pleiotropic effect and linkage disequilibrium^81^. Mota et al.^25^ observed that FE-related traits show pleiotropic genomic regions with significant contributions to explain feed efficiency in Nellore cattle. Hayashi and Iwata^82^ reported a gain in prediction accuracy from a multi-trait against single-trait genomic prediction. These authors pointed out that MTGBLUP is more advantageous for traits with low heritability that are genetically correlated with a high heritability trait. Karaman et al*.*^50^ observed that multi-trait models improved prediction accuracy compared to single-trait models.

Various authors observed that multi-trait models lead to higher prediction accuracies with a limited number of phenotypic information than the traditional single-trait genomic model, particularly when traits considered in the model are genetically correlated^23,50,75,83,84^. We have found that the MTGBLUP was highly effective for enhancing predictive ability, possibly by taking advantage of the moderate to strong genetic correlations between the traits. In summary, we compared machine learning and multi-trait GBLUP model with traditional parametric methods for predicting feed efficiency-related traits in Nellore cattle, and we observed that SVR and MTGBLUP models showed similar accuracies but that they outperformed the other approaches. Using the SVR method, improvements in predictive ability might be related to considering radial basis functions to capture nonlinear relationships between SNP markers, which better explain the trait variability^44^. However, MTGBLUP, in which marker effects are assumed to follow normal distributions with the same variance, takes advantage of jointly modeling multiple characteristics through their correlations, providing increased predictive quality^22,82,85^.

## Conclusions

In this study, we assessed the performance of two machine learning approaches (multi-layer neural network–MLNN, and support vector regression—SVR) and MTGBLUP compared to the traditional methods (STGBLUP and Bayesian regression) in terms of their prediction accuracy of feed efficiency (FE)-related traits in Nellore cattle. We found that the SVR, MLNN, and MTGBLUP models outperformed the SGBLUP and Bayesian regression models. Prediction accuracies obtained with SVR and MTGBLUP were similar to each other, with less unbiased predictions, indicating that both approaches are suitable for genomic prediction of FE-related traits in Nellore cattle, mainly by their easy implementation.

### Supplementary Information

Supplementary Figures.

## Data Availability

The data used in this study is a part of an experimental breeding program, and it was shared with the authors through a collaboration agreement. Therefore, the dataset is not available to the public, but it can be accessed by contacting the corresponding authors upon a reasonable request.
