# Supplementary material for: Benchmarking machine learning and parametric methods for genomic prediction of feed efficiency-related traits in Nellore cattle
Source: Sci Rep. 2024 Mar 17;14:6404. doi: 10.1038/s41598-024-57234-4 (PMC10944497; doi:10.1038/s41598-024-57234-4)
Supplement: Supplementary file 1 — Supplementary Figures. [file 41598_2024_57234_MOESM1_ESM.docx]

# Benchmarking machine learning and parametric methods for genomic prediction of feed efficiency-related traits in Nellore cattle

Lucio F. M. Mota^1*^, Leonardo M. Arikawa^1^, Samuel W. B. Santos^1^, Gerardo A. Fernandes Júnior^1^, Anderson A. C. Alves^1^, Guilherme J. M. Rosa^2^, Maria E. Z. Mercadante^3,4^, Joslaine N. S. G. Cyrillo^3^, Roberto Carvalheiro^1,4^, Lucia G. Albuquerque^1,4*^

^1^São Paulo State University (UNESP), School of Agricultural and Veterinarian Sciences, Via de Acesso Prof. Paulo Donato Castelane, Jaboticabal - SP, 14884-900, Brazil.

^2^Department of Animal and Dairy Sciences, University of Wisconsin-Madison, Madison, WI, USA

^3^Institute of Animal Science, Beef Cattle Research Center, Sertãozinho - SP, 14174-000, Brazil.

^4^National Council for Science and Technological Development, Brasilia - DF, 71605-001, Brazil

*Corresponding authors

Lucio F. M. Mota ([flaviommota.zoo@gmail.com](mailto:flaviommota.zoo@gmail.com)) and Lucia G. Albuquerque ([galvao.albuquerque@unesp.br](mailto:galvao.albuquerque@unesp.br))

## Supplementary Information

Supplementary Figure S1. Principal component analysis of animals based on the first two principal components using the SNP information to evaluate the extent of the population structure in the IZ population (NeC – Nelore Control; NeS – Nelore Selection; and NeT – Nelore Traditional).


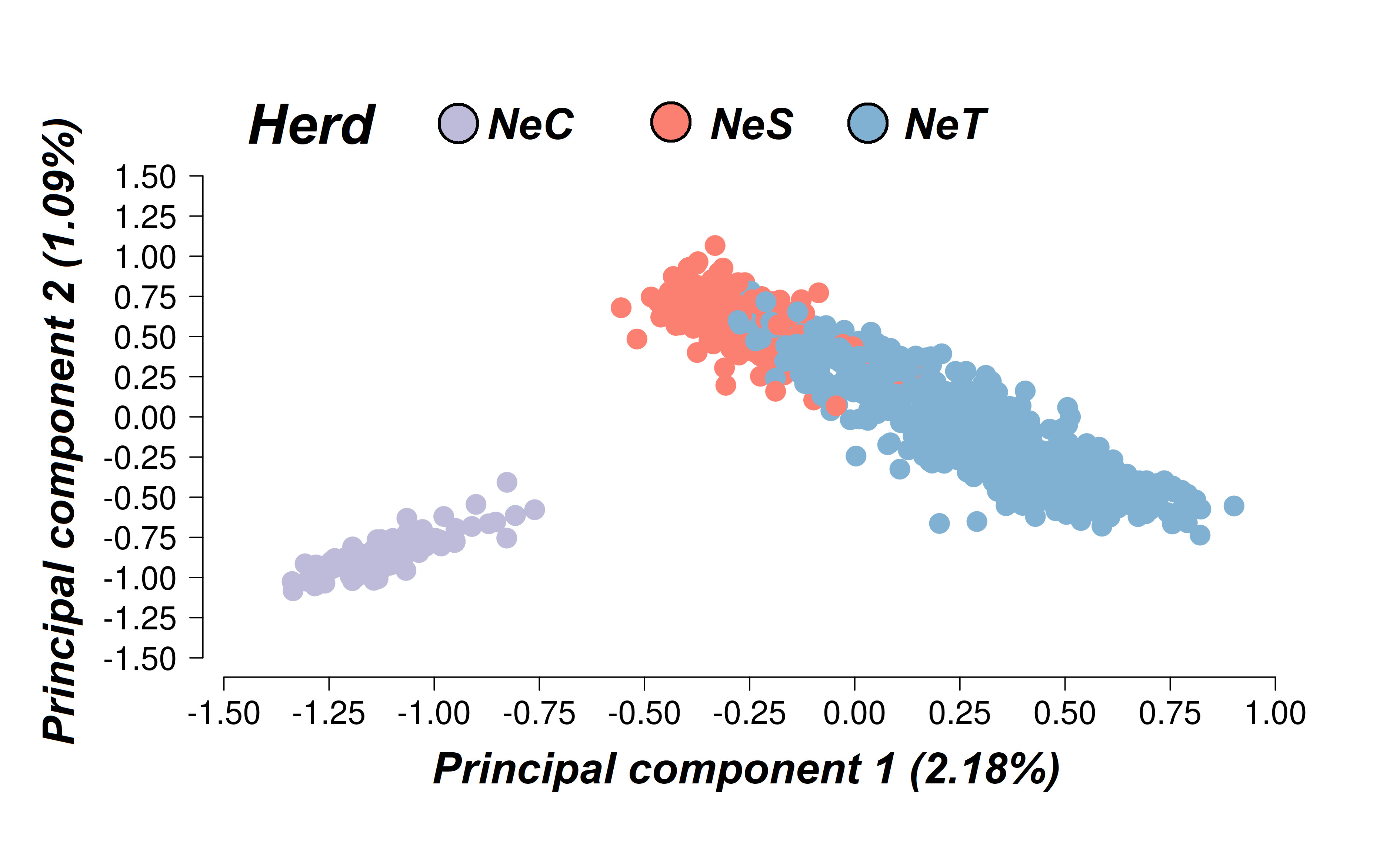

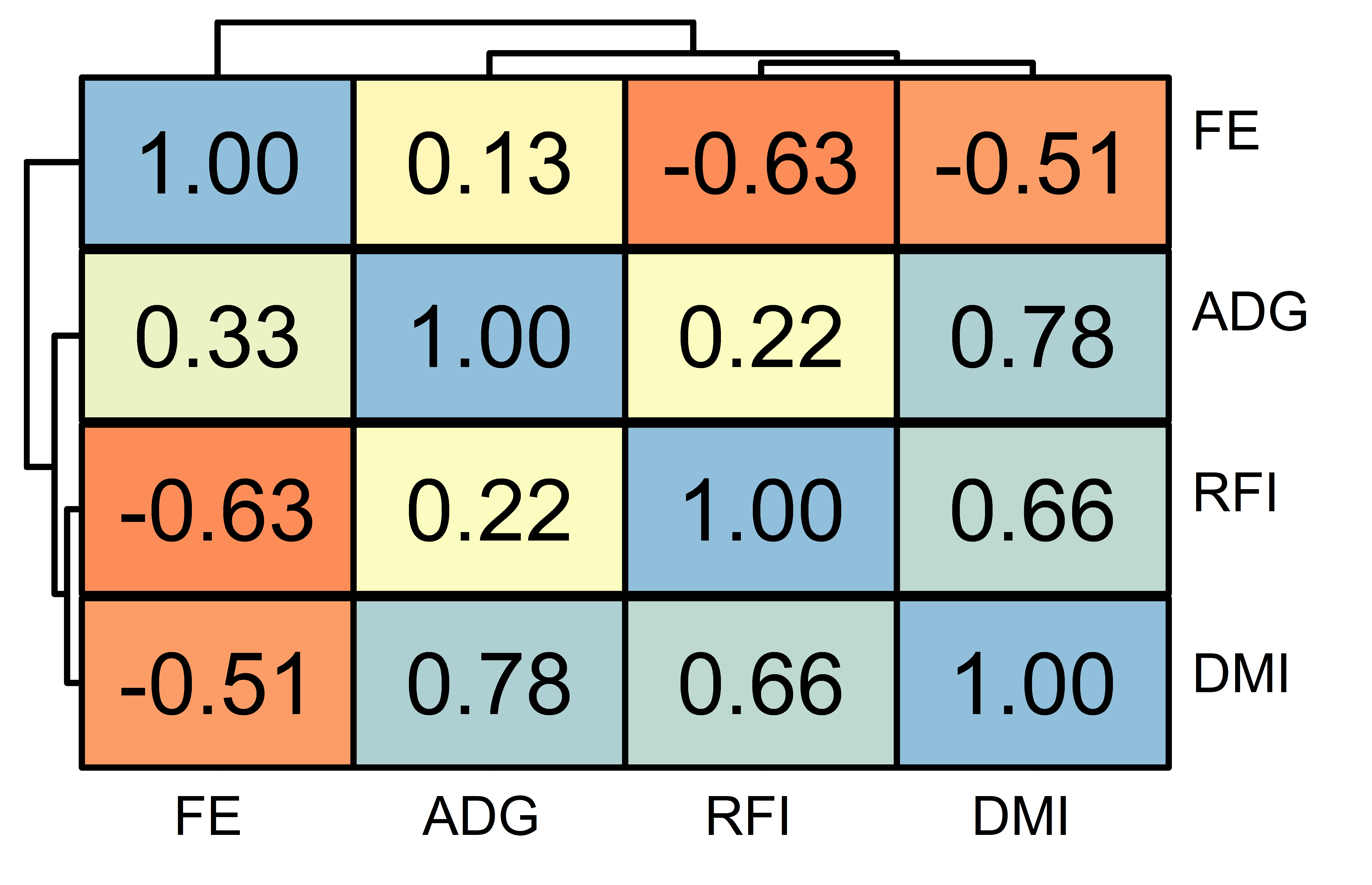


Supplementary Figure S2. Genetic correlation between feed efficiency-related traits in *IZ population* and Ward’s hierarchical clustering of genetic correlation for feed efficiency-related traits. ADG – average daily gain; FE – feed efficiency; RFI – residual feed intake; DMI – dry matter intake.


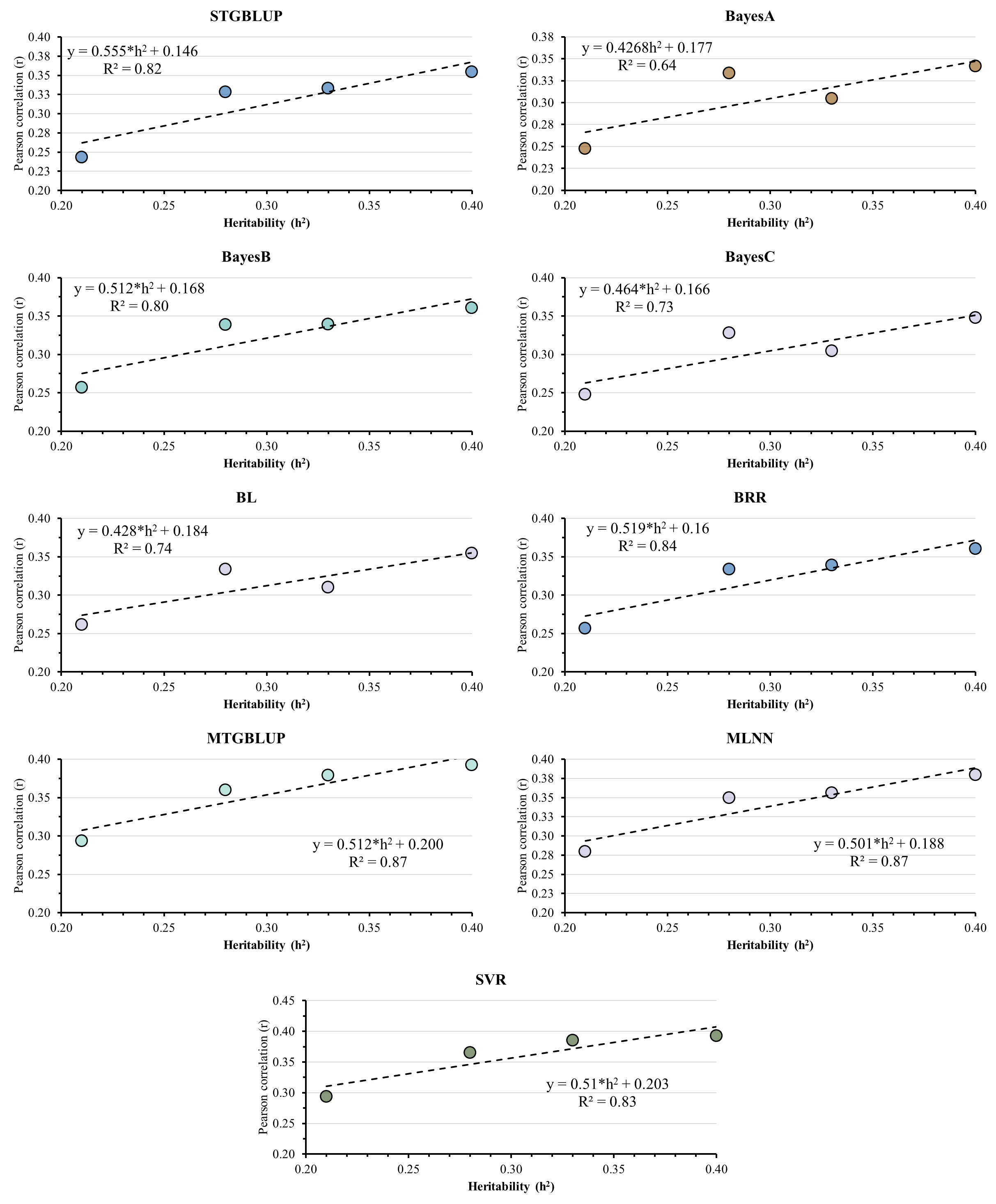


Supplementary Figure S3. Relationship between Pearson correlation of adjusted phenotype and genomic breeding value obtained using forward validation and heritability estimates for feed efficiency related traits. STGBLUP – single trait GBLUP, BayesA – Bayesian A, BayesB – Bayesian B, BayesC – Bayesian C, BL – Bayesian Lasso, BRR – Bayesian ridge regression, MTGBLUP – multi-trait GBLUP, MLNN – Multilayer neural networks, and SVR – support vector machine regression using a radial basis kernel.


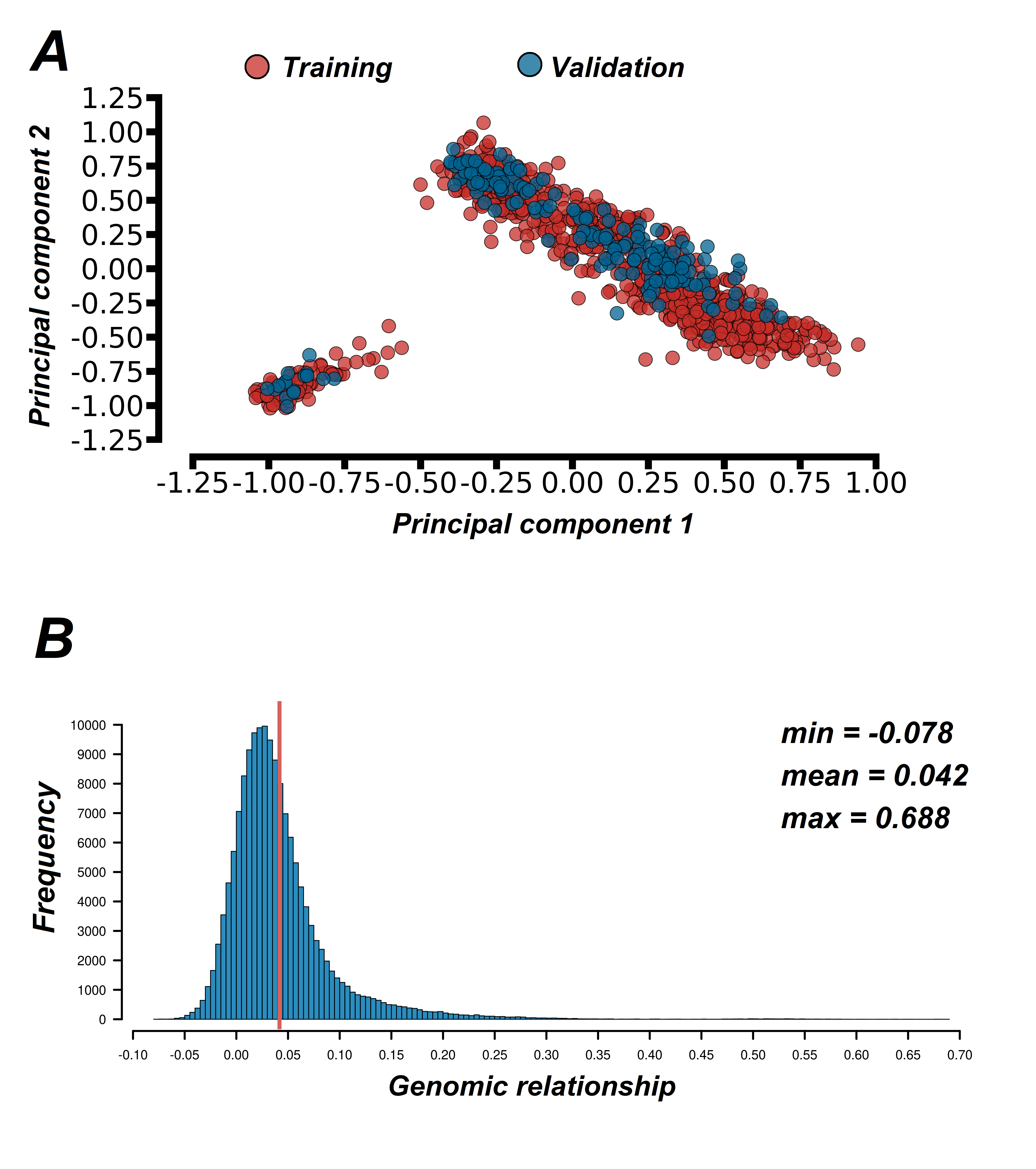


Supplementary Figure S4. Distribution of animals in training (red) and validation (blue) population for forward validation scheme, obtained by PCA based on the genomic matrix [A]. Genomic relationships among animals in training and validation used to assess the predictive ability for feed efficiency related traits in Nellore cattle [B]. The red line indicates the mean of genomic relationship among animals in validation and training set.
